# Pathological, Bacteriological and Virological Findings in Sudden and Unexpected Deaths in Young Dogs

**DOI:** 10.3390/ani10071134

**Published:** 2020-07-03

**Authors:** Giuseppe Piegari, Lorena Cardillo, Flora Alfano, Lucia Vangone, Valentina Iovane, Giovanna Fusco

**Affiliations:** 1 Department of Veterinary Medicine and Animal Production, Unit of Pathology, University of Naples Federico II, 80137 Naples, Italy; 2 Istituto Zooprofilattico Sperimentale del Mezzogiorno, Portici, 81100 Naples, Italy; lorena.cardillo85@gmail.com (L.C.); flora.alfano@cert.izsmportici.it (F.A.); lucia.vangone@izsmportici.it (L.V.); giovanna.fusco@izsmportici.it (G.F.); 3 Department of Pharmacy, University of Salerno, 84084 Fisciano, Italy; viovane@unisa.it

**Keywords:** post-mortem microbiology, veterinary forensic pathology, sudden death, young dogs

## Abstract

**Simple Summary:**

“Sudden death” has been defined by the World Health Organization as a non-violent, unexpected death occurring less than 24 h from the onset of symptoms. The causes of sudden death have been widely investigated in human forensic medicine. In contrast, few studies have been reported in the veterinary literature. This study aimed to investigate the frequency of sudden deaths in young dogs in different age ranges. A secondary aim was to collect information regarding clinical symptoms, and pathological and microbiological findings related to sudden death in young dogs. The results of the present study demonstrate that the highest frequency of sudden death occurs in animals in an age range from 10 days to 1 month and from 6 to 12 months. The most frequently observed clinical symptoms in cases of sudden death were acute respiratory symptoms. Furthermore, *Canine parvovirus type 2*, *E. coli*, *Canine Distemper Virus, Clostridium perfringens type A, and Pasteurella* spp. were the main causes of death observed in the present study. The results reported in the present study could provide a reference basis to better investigate sudden death in veterinary clinical practice.

**Abstract:**

In human medicine, “sudden death” has been defined by the World Health Organization (WHO) as a non-violent, unexpected death occurring less than 24 h from the onset of symptoms. The aims of this study were: (1) to estimate the proportional mortality ratio for “sudden and unexpected death” (SUD) in young dogs; (2) to investigate the pathological and microbiological findings in SUD cases in young dogs. For these purposes, a retrospective study of a total of 145 cases of young dead dogs was performed. For each case, we collected information about the age, medical history and the gross and microbiological findings of the animals. The results of this study found 21 cases of SUD. The most frequently observed clinical symptoms in the cases of sudden death were acute respiratory symptoms, followed by acute gastroenteric symptoms, non-specific symptoms and neurological symptoms. The evaluation of necropsy reports allowed us to observe enteritis in 18 out of 21 cases and pneumonia in seven out of 21 cases. Viral infection with *Canine parvovirus type 2* was the most common cause of SUD observed. These results could provide a valuable tool for the investigation of sudden death in young dogs.

## 1. Introduction

In human medicine, “sudden death” has been defined by the World Health Organization (WHO) as a non-violent, unexpected death occurring less than 24 h from the onset of symptoms [1]; in particular, the term “sudden and unexpected infant death” (SUID) is used to describe deaths that occur relatively suddenly and unexpectedly in children less than 1 years old [2,3]. Infections are reported in the literature as an important cause of SUID, followed by metabolic or molecular disorders [2,3,4,5]. The main pathogens reported in SUID cases are as follows: *Staphylococcus aureus*, *Escherichia coli*, *Streptococcus pyogenes*, *Streptococcus agalactiae*, *Streptococcus pneumoniae*, *Group B Streptococci (GBS), Respiratory Syncytial Virus (RSV), Cytomegalovirus (CMV) and Adenovirus* [2,6,7]. However, a broad range of pathogens has been reported in the literature as being causes or a co-factors in SUID, such as *Parvovirus B19, Epstein-Barr virus, Influenza A virus* and *Mycobacterium tuberculosis* [2,6,8,9]. Furthermore, recent studies reported the relatively benign Coxsackie virus A16 as a possible contributing factor in SUID in humans [10]. For these reasons, the current SUID autopsy protocol in the UK and the international guidelines advocate for a multidisciplinary approach to the investigation of all cases of SUID, which should be based not only on the findings of the macroscopic examination, but also on a broad range of ancillary investigations, such as bacteriological and virological analyses [11,12]. Sudden infant death syndrome (SIDS) is considered to be a sub-class of SUID, in which the cause of death remains unexplained even after the forensic necropsy, ancillary tests and evaluation of the anamnestic data and crime scene analysis [1,2]. Indeed, among the cases of SUID, only 20% have a clear cause, while most cases remain unexplained and are categorized as SIDS [1,5,13]. Although the cause is unknown, specific genetic mutations or mild infections could be involved in the genesis of the syndrome [5]. Mild infections have been suggested to play a key role, as demonstrated by altered levels of immunoglobulin or cytokine and the high frequency of mild tracheal infections commonly observed during post-mortem examinations of the subjects with a final diagnosis of SIDS [5]. Although, in human medicine, the concept of sudden death, SUID and SIDS has been well defined by the WHO, in veterinary medicine, a universal definition is lacking. Some authors have defined sudden death in animals as death that occurs in a few minutes or several hours, due to pre-existing disease or a functional disorder [14]. However, in the opinion of the authors, this definition should be avoided, because it lacks a well-defined temporal reference range. In contrast, even if not yet validated in veterinary medicine, the WHO definition provides an important temporal reference range useful for the identification of cases of sudden death in veterinary clinical practice.

Over the last few years, many studies have investigated the cause of death in animals. In particular, infectious diseases that affect the gastrointestinal system are reported to be the main cause of death in puppies and young dogs [15,16]. In contrast, neoplastic diseases appear to be the prevalent cause of death in adult dogs [15]. Among the infections, *canine parvovirus type 2* (CPV-2) is reported to be one of the most common and important causes of morbidity and mortality in young dogs [17,18]. Moreover, this virus is considered to be an important pathogen responsible for acute gastroenteritis and myocarditis in dogs [17,18]. However, with regards to sudden and unexpected deaths, despite the underlying causes having been sporadically investigated in dogs [19], to the best of our knowledge, no study has evaluated the microbiological findings in cases of sudden death in young dogs. In light of these observations, the aims of this study were as follows: (1) to estimate the proportional mortality ratio (PMR) for “sudden and unexpected death” in puppies and young dogs; (2) to investigate the pathological, bacteriological, and virological findings in sudden and unexpected death in young dogs; and (3) to introduce a standardized microbiological protocol for the diagnostic investigation of cases of sudden death in veterinary medicine

## 2. Materials and Methods

### 2.1. Study Design

An observational retrospective study of a total of 145 cases of young dead dogs, consecutively presented by veterinary practitioners, owners, or law enforcement to the “Istituto Zooprofilattico del Mezzogiorno” (IZSM) of Portici city, Southern Italy, was performed over a 3-year period (2015–2017). The submission forms were collected to obtain information about the medical history and age of the animals. On the basis of the medical history, the animals were divided into groups as follows:

- (Sudden and unexpected death group—SUD group): dogs with a clinical diagnosis of sudden and unexpected death. According to the WHO, sudden and unexpected death (SUD) cases were considered to be a non-violent and unexpected death that occurs less than 24 h after the onset of symptoms;
- (Expected death group—ED group): dogs without a clinical history of sudden and unexpected death

On the basis of age, the available data were categorized as follows: (Group 1) 10 days–4 weeks; (Group 2) 4 weeks–6 weeks; (Group 3) 6 weeks–2 months; (Group 4) 2–3 months; (Group 5) 3–6 months; (Group 6) 6–12 months. Each examined case was subjected to a complete necropsy and bacteriological and virological investigations; however, for the purposes of this study, only the necropsy and microbiological reports from animals in the SUD group were included. Furthermore, the microbiological investigations were restricted to molecular tests for the virological analysis, and microbiological cultures for the bacteriological examinations. In all assessed cases, molecular tests were performed using a real-time polymerase chain reaction assay (RT-PCR) for *canine parvovirus (CPV), canine coronavirus, canine adenovirus, herpesvirus, and canine distemper virus*. Furthermore, in all cases positive for *canine parvovirus type 2*, multiplexed PCR panels were used to distinguish between wild-type and vaccine CPV-2 and to identify the pathogen subtype (CPV-2a; CPV2b; CPV 2c) [20]. The analyzed samples included the liver, lung, kidney, spleen, heart, brain and intestine. Microbiological results and necropsy reports were both extracted from the IZSM information system (SIGLA). Animals that tested positive in the toxicological investigations, or that had died from trauma, were excluded from the study.

### 2.2. Necropsy Protocol

All necropsies were performed in the necropsy room of the “Istituto Zooprofilattico Sperimentale del Mezzogiorno” (IZSM), Portici, Italy, with a standard necropsy protocol [21]. All SUD cases were stored at 4 °C before necropsy. The period between death and necropsy was between 12 and 36 h. During the necropsy, according to internal institute protocol, all samples were taken in rigorous asepsis conditions using sterile instruments and transported to the laboratory of microbiology. Furthermore, to obtain uncontaminated specimens, a sterilization of the body and organs surfaces was performed before sampling. Finally, the mean time between sample collection and transport to the reference laboratory was under 3 h.

### 2.3. Analytical Validation of the Results

For each case of sudden and unexpected death, the clinical history, necropsy report and microbiological findings were reviewed, and the final cause of death was categorized as “explained” or “unexplained”. However, since determining the pathological significance of the microorganisms isolated during necropsy is often difficult, as has been frequently reported in the literature [22,23]. For the purposes of this study, viruses detected by PCR were considered to be the cause of death, only when associated with the typical macroscopic changes observed during the anatomopathological examination. In addition, the bacteriological and virological findings were interpreted considering a broad range of variables, such as the location of pathogen detection, the capacity of pathogens for virulence, the correlation with injuries observed during the necropsy, the multisite location of the pathogens, the age of the dog and the composition of the normal flora.

### 2.4. Statistical Analysis

The frequencies of sudden and unexpected death (SUD), expected death (ED), and total deaths (SUD + ED) were evaluated and stratified by age classes. Furthermore, we estimated the proportional mortality ratio (PMR) for “SUD” in each assessed age group. The Chi-square test was used to assess differences in the distributions of ED and SUD among age groups.

## 3. Results

Out of the 145 examined reports, we found 21 cases of SUD and 124 cases of ED during the 3-year study period. The PMR of SUD was therefore 14.48%, while the ED was 85.52%. Furthermore, the Chi-square test showed a significant difference in the frequencies of ED and SUD among the assessed age groups (*p* < 0.05). All SUD cases were submitted by Italian veterinary practitioners. The highest frequencies of expected death were observed in animals in Group 2 (100% of the cases), Group 3 (87.7% vs. 12.5%), Group 4 (97.2% vs. 12.8%) and Group 5 (93.3% vs. 6.7%). In contrast, the highest frequencies of SUD were found in animals in Group 1 (58.8% vs. 41.2%) and Group 6 (37.5% vs. 62.5%). Table 1 summarizes the frequencies and percentages of SUD and ED and the frequency of total deaths (SUD + ED) stratified by age classes. Overall, of the 21 SUD cases, 10 out of 21 (47.61%) dogs were less than 4 weeks old (Group 1), 0 out of 21 were between 4 weeks and 6 weeks old (Group 2), five out of 21 (23.8%) were between 6 weeks and 2 months old (Group 3), one out of 21 (4.76%) was between 2 and 3 months old (Group 4), two out of 21 (9.51%) were between 3 and 6 months old (Group 5), and three out of 21 (14.28%) were between 6 months and 1 year old (Group 6).

### 3.1. Clinical Background and Gross Findings

**Figure 1:**
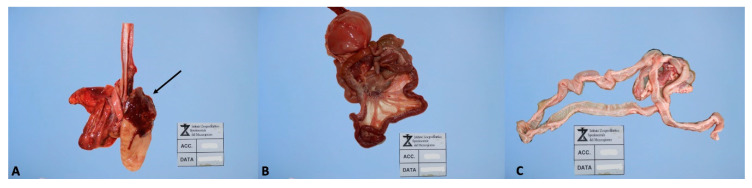
Pathological lesions in cases of sudden death. (**A**): pneumonia (arrows) (**B**): haemorrhagic gastroenteritis (**C**): catarrhal enteritis.

The most frequently observed clinical symptoms in cases of sudden death were as follows: acute respiratory symptoms in 12 out of 21 cases, followed by acute gastroenteric symptoms (a single or few episodes of vomiting or diarrhea) in six out of 21 cases, neurological symptoms in one case, and finally, non-specific symptoms in two out of 21 cases. The evaluation of necropsy reports allowed us to observe haemorrhagic gastroenteritis in 12 out of 21 cases, pneumonia in seven out of 21 cases, and catarrhal enteritis in five out of 21 cases (Figure 1). Pulmonary oedema or multiorgan congestion were also observed in 19 out of 21 cases.

### 3.2. Microbiological Examination

In all animals dead for sudden and unexpected death, virological investigations were performed with a panel of viruses tested by PCR (*Canine parvovirus, Canine coronavirus, Canine adenovirus, Canine herpesvirus* and *Canine distemper virus*), and a bacteriological examination was performed with microbiological cultures. The retrospective analysis showed positive microbiological results in 18 out of 21 cases (Table 2).

However, for the purposes of this study, the microbiological findings were interpreted considering a broad range of variables. In particular, the location of pathogen detection, the capacity of pathogens for virulence and the correlation of that microorganisms with the observed macroscopic injuries were the most important parameters assessed in this study. Therefore, after the review of the necropsies and microbiological reports, the detected pathogens were considered the main cause of death in only 14 out of 21 cases. In particular, among the evaluated cases, the main cause of death was viral infection with *Canine parvovirus type 2* (8/21), followed by viral infection with *Canine parvovirus type 2,* and co-infection with *E. coli* (2/21), bacterial co-infection with *Clostridium perfringens* type A and *E. coli* (2/21) and viral and bacterial co-infection with the *Canine distemper virus* and *Pasteurella* spp. (2/21). Finally, in seven out of 21 cases, the microbiological results did not explain the injuries observed during the necropsy. Therefore, the causative agent of infection was considered undetermined after the microbiological examination. Table 3 summarizes the clinical backgrounds, pathological findings, microbiological results and causes of death of the cases of sudden death.

## 4. Discussion

In human forensic pathology, the autopsy for cases of SUID are primarily performed according to the “Kennedy Report” [11]. This protocol and the published international guidelines advocate a multidisciplinary approach to investigations of all cases of SUID, which should be based not only on the findings of the post-mortem macroscopic examination, but also on a broad range of ancillary investigations, such as bacteriological and virological analyses. Although a broad range of tests have been proposed in cases of SUID in human forensic medicine, in the present study, we focused on the pathological and post-mortem microbiology findings in cases of sudden death in young dogs. The results of this study show a low frequency of sudden deaths in young dogs, accounting for 14.48% of the total observed deaths. Furthermore, the Chi-squared test showed a significant difference in the frequencies of ED and SUD among the assessed age groups (*p* < 0.05). In particular, the highest frequency of sudden death was observed in dogs younger than 4 weeks old. In contrast, the highest frequency of ED was observed in animals in Groups 2–5. This difference could be due to the immaturity of the immune system of puppies younger than 6–12 weeks of age [24]. Indeed, the endotheliochorial placentation of this species is relatively impenetrable to the transfer of maternal immunoglobulin [24]. Thus, the immune protection of the puppies during the first weeks of life depends on the ingestion of maternal colostrum antibodies (MCA) [24]. In the absence of the passive transfer of MCA, newborn puppies are only able to develop an immune response to antigens at 2–3 weeks of age. Therefore, any delay in colostrum intake or reduction of colostrum ingestion leads to a reduction in the immune protection of the animals [24,25,26]. Under these conditions, viruses or bacteria can replicate and spread quickly, leading to the death of the puppies, without the development of characteristic symptoms. Furthermore, congenital malformation or maternal malnutrition could be considered additional causes of sudden death in this age range. In contrast, after 2–3 weeks of age, the immune system of puppies, although immature, is able to develop a mild immune response against pathogens, avoiding the rapid spread of the pathogens and allowing the development of characteristic symptoms of the pathology. In addition, in our study, the most frequently observed gross injuries in cases of sudden death were haemorrhagic gastroenteritis in 12 out of 21 cases, pneumonia in seven out of 21 cases, and catarrhal enteritis in five out of 21 cases. Pulmonary oedema or multiorgan congestion were also observed in 19 out of 21 cases. Intra-abdominal and respiratory lesions have been previously reported in the literature as two important causes of sudden and unexpected infant death in human forensic pathology [27]. Indeed, respiratory tract lesions, although mild, can easily lead to serious complications and sudden death of the subjects [27]. Similarly, acute gastroenteric lesions can cause severe dehydration and serum electrolyte disturbance, which have the potential to cause sudden and unexpected death in children [27]. Overall, in our study, positive microbiological results were observed in 18 out of 21 cases. However, as frequently highlighted in the human literature, the isolation of pathogens in cases of sudden death does not necessarily imply a correlation between those pathogens and the death. In particular, this correlation must be confirmed by the observation of severe and specific injuries during the anatomopathological examination [6]. Therefore, after the review of the necropsy findings, detected pathogens were considered the main cause of death in only 14 out of 21 cases, while in the remaining seven cases, the microbiological results did not explain the injuries observed during the necropsy. The negative findings observed in our study could suggest: (1) a non-infectious cause of death of the assessed animals; or (2) a death due to viruses or other pathogens not detected by the virological panel in use in this study. Indeed, there are a wide range of viruses that are potentially pathogenic in young dogs, including both DNA and RNA viruses. However, our virological panel was limited to the detection of the following five specific viruses: *canine parvovirus, canine coronavirus, canine adenovirus, canine herpesvirus* and *canine distemper virus.* With regard to the positive results, viral infection due to *canine parvovirus type 2* (wild type) was the most common cause of death observed in our study. Overall, *CPV-2* is a causative agent of acute gastroenteritis and myocarditis [17,18]. Furthermore, it is reported in the literature to be one of the most common and important causes of morbidity and mortality in young dogs [17,18]. Usually, the clinical symptoms of the infection are as follows: anorexia, depression, lethargy and fever, followed by vomiting and diarrhea [17,18,28]. However, it is also reported to be a cause of sudden cardiac death in puppies between 4 and 8 weeks of age [17,29,30]. Interestingly, we also observed two cases of sudden death due to *Canine distemper virus* and *Pasteurella* spp. co-infection. *Canine distemper virus* is a member of the genus *Morbillivirus*, which can cause a large variety of disorders in dogs including rhinitis, pneumonia, demyelinating leukoencephalitis, necrotizing bronchiolitis and enteritis [31]. *Canine distemper virus* is not reported in the veterinary literature as a cause of sudden death in animals. However, *Canine distemper virus* causes immunosuppression by targeting cells that express the CD150 protein (signaling lymphocyte activation molecule (SLAM)) [31]. Previous studies showed that this immunosuppression favors secondary infections caused by pathogens, such as *Bordetella bronchiseptica* or *C. piliforme* [32]. Therefore, it is possible to suppose that opportunistic pathogens could complicate a sub-clinical *Canine distemper virus* infection, replicating and spreading quickly, and leading to the death of the puppies without the development of characteristic symptoms. With regards to the bacteriological examination, the most common isolated bacteria were *C. perfringens* type A (6/19) and *E. coli* (6/19). However, they were considered the cause of death in only two cases. Indeed, *C. perfrigens* and *E. coli* are considered normal components of canine intestinal flora [33,34]. Similarly, the alpha toxin gene of *C. perfringens* may be found in asymptomatic dogs as part of the normal intestinal microflora [33]. However, in some cases, *E. coli* can cause pleuro-pneumonitis [35], gastroenteritis [36], urogenital infections, cholangitis, cholangiohepatitis and septicaemia [37] in both humans and other animals. Similarly, *C. perfringens* type A has been associated with gastro-enteric disease, such as haemorrhagic enteritis in dogs and abomasitis in ruminants [38,39,40]. Furthermore, this bacterium has been reported in the literature as a cause of sudden and unexpected death in dogs [39]. Unfortunately, no specific test for the diagnosis of enteritis due to *C. perfringens* is described in the literature [40]. Thus, generally, the clinical signs, the pathological findings, the microbiological analysis, and the absence of other pathogens must be examined before confirming the diagnosis [40]. In our case, the multisite locations of the pathogens, the absence of other viruses or bacteria and the specific anatomopathological findings of haemorrhagic enteritis supported the diagnosis of enteritis due to *C. perfringens* and *E. coli* as the final cause of death.

Finally, this study allowed the detection of a wide range of pathogens that, after the review of the necropsy and microbiological reports, were not considered the main cause of death of the animals, such as *Canine Adenovirus, Rotavirus*, *Streptococcus sanguinis*, *Streptococcus dysgalactiae* and, in some cases, *E. coli* and *C. perfringens*. Therefore, further studies will be needed to evaluate the possible contributions of these pathogens to cases of sudden and unexpected death in young dogs.

## 5. Conclusions

Sudden death is an uncommon cause of death in young dogs. However, the high frequencies of viruses and bacteria detected in our study highlights the importance of performing complete bacteriological and virological analyses in all cases of sudden death in young dogs. The results of this study suggest that our PCR panel combined with a bacteriological analysis could facilitate the rapid detection and type-specific identification of the pathogenic causes or co-factors of death in most cases of sudden death in young dogs. Finally, these results could provide a valuable epidemiological tool for the investigation of sudden death in young dogs.

## Figures and Tables

**Table 1:** Frequency and percentage of sudden and unexpected deaths, expected deaths and the frequency of total deaths stratified by age groups.

Age Group	Sudden Death	Expected Death	Total Deaths
Group 1	10 (58.8%)	7 (41.2%)	17
Group 2	0	14 (100%)	14
Group 3	5 (12.5%)	35 (87.5%)	40
Group 4	1 (2.8%)	35 (97.2%)	36
Group 5	2 (6.7%)	28 (93.3%)	30
Group 6	3 (37.5%)	5 (62.5%)	8
Total	21	124	145

**Table 2:** Viruses or bacteria detected in cases of sudden and unexpected death.

Pathogen	NO. of Cases
*Canine Parvovirus*	10
*E. Coli*	7
*Clostridium perfringens type A*	6
*Adenovirus*	3
*Canine Distemper Virus*	2
*Streptococcus sanguinis*	2
*Pasteurella* spp.	2
*Streptococcus dysgalactiae*	1
*Rotavirus*	1

**Table 3:** Microbiological and anatomopathological findings of the studied dogs.

Group	Sex	Clinical Background	Pathological Findings	Virological Examination	Bacteriological Examination	Cause of Death
1	M	Acute respiratory insufficiency	Visceral congestion, pulmonary edema, hemorrhagic gastroenteritis	*Canine parvovirus type 2a* (wild type)—detected in the lung, liver, heart, brain, and intestine	*Streptococcus dysgalactiae*—isolated in the lung	Viral infection
	M	Acute respiratory insufficiency	Visceral congestion, pulmonary edema, severe broncho-pneumonia, catarrhal enteritis	No viruses detected	*Streptococcus sanguinis*—isolated in the intestine	Undetermined: severe pneumonia due to unexplained causes
	M	Acute respiratory insufficiency	Visceral congestion, pulmonary edema, severe broncho-pneumonia, catarrhal enteritis	No viruses detected	*Streptococcus sanguinis*—isolated in the intestine	Undetermined: severe pneumonia due to unexplained causes
	F	Acute respiratory insufficiency	Visceral congestion, multifocal pulmonary hemorrhages, hemorrhagic enteritis	*Canine parvovirus type 2b and 2c* (wild type)—detected in the lung, liver, spleen, heart, and intestine	*Clostridium perfringens*, Detection of *Clostridium perfringens* alpha toxin—isolated in the intestine	Viral infection
	M	Acute respiratory insufficiency	Lobar pneumonia, pulmonary edema, catarrhal enteritis	*Canine distemper virus—*detected in the lung, liver*,* and brain	Pasteurella spp.—detected in the lung	Viral and bacterial infection
	F	Acute respiratory insufficiency	Visceral congestion, multifocal pulmonary hemorrhages, hemorrhagic enteritis	*Canine parvovirus type 2b and 2c* (wild type)—detected in the lung, liver, brain, heart, and intestine	*E. coli, Clostridium perfringens*, Detection of *Clostridium perfringens* alpha toxin—isolated in the intestine	Viral infection
	M	Acute respiratory insufficiency	Visceral congestion, pulmonary edema, focal broncho-pneumonia, hemorrhagic gastroenteritis	*Canine distemper virus—*detected in the lung, liver*,* and brain	*Pasteurella* spp.—detected in the lung	Viral and bacterial co-infection
	M	Acute respiratory insufficiency	Visceral congestion, pulmonary edema, hemorrhagic gastroenteritis	No viruses detected	No bacteria detected	Undetermined
	M	Acute respiratory insufficiency	Visceral congestion, pulmonary edema, severe broncho-pneumonia	No viruses detected	No bacteria detected	Undetermined
	M	Acute respiratory insufficiency	Visceral congestion, hemorrhagic enteritis	*Canine parvovirus type 2a* (wild type)—detected in the heart, spleen, and intestine	No bacteria detected	Viral infection
3	M	Sialorrhea, unilateral eye swelling, muscle stiffness, a single episode of vomiting	Visceral congestion, pulmonary edema, hemorrhagic enteritis	No virus detected	*E. coli; Clostridium perfringens* Detection of *Clostridium perfringens* alpha toxin—detected in the intestine and lung	Bacterial infection
	M	Neurological symptoms	Visceral congestion, bi-lateral pneumonia, pulmonary edema segmental catarrhal enteritis	Rotavirus (detected in the intestine)	*E. coli*—isolated in the intestine	Undetermined: severe pneumonia due to unexplained causes
	F	Acute gastrointestinal symptoms	Visceral congestion, hemorrhagic enteritis, focal pneumonia	*Canine parvovirus type 2a*, (wild type)—detected in the lung, liver, and intestine; *Adenovirus*—detected in intestine	*Clostridium perfringens* Detection of *Clostridium perfringens* alpha toxin—isolated in the intestine	Viral infection
	F	Vomiting	Visceral congestion, hemorrhagic enteritis, focal pneumonia	*Canine parvovirus type 2a* (wild type)—detected in the lung, liver, heart, and intestine	*E. coli*—isolated in intestine and lung	Viral and bacterial co-infection
	M	Vomiting	Visceral congestion, pulmonary congestion, enteritis, abdominal, thoracic and pericardial effusion, multifocal pulmonary hemorrhage	No virus detected	*E. coli*—isolated in intestine	Undetermined: insufficient findings to explain death
4	F	Acute respiratory insufficiency	Multifocal hemorrhage, abdominal, thoracic and pericardial effusion, hemorrhagic enteritis	No virus detected	*E. coli*—detected in the liver, lung, and intestine *Clostridium perfringens* Detection of *Clostridium perfringens* alpha toxin—detected in the intestine and lung	Bacterial infection
5	M	Acute respiratory insufficiency	Pulmonary congestion, segmental catarrhal enteritis	*Canine parvovirus type 2a* (wild type)—detected in lung, liver, and intestine	*E. coli*—isolated in the intestine	Viral infection
	F	A single episode of diarrhea	Pulmonary congestion, pulmonary edema, segmental hemorrhagic enteritis	*Canine parvovirus type 2a* (wild type)—detected in lung, liver, intestine, and spleen *Adenovirus*—detected in the lung	No bacteria detected	Viral infection
6	F	Lack of appetite	Thoracic effusion, visceral congestion, multifocal hemorrhage, severe hemorrhagic enteritis	*Canine parvovirus type 2a* (vaccinal) and *2c* (wild type)—detected in lung, liver, intestine, and spleen *Canine distemper virus-detected in lung* *Adenovirus—detected in lung*	No bacteria detected	Viral infection
	F	Lack of appetite and fever for 12 h	Multifocal hepatic necrosis, hemorrhagic enteritis	*Canine parvovirus type 2b* (wild type)—detected in the lung, liver, brain, and intestine	*E. coli. Clostridium perfringens*, Detection of *Clostridium perfringens* alpha toxin (isolated in lung, liver, and intestine)	Viral and bacterial infection
	F	Single episode of diarrhea	Congestion of the spleen, abdominal effusion	No viruses detected	No bacteria detected	Undetermined: insufficient findings to explain death
